# Natural Small Molecules Targeting NF-κB Signaling in Glioblastoma

**DOI:** 10.3389/fphar.2021.703761

**Published:** 2021-08-27

**Authors:** Md. Sahab Uddin, Md. Tanvir Kabir, Abdullah Al Mamun, Md. Shahid Sarwar, Fatema Nasrin, Talha Bin Emran, Ibtesam S. Alanazi, Abdur Rauf, Ghadeer M. Albadrani, Amany A. Sayed, Shaker A. Mousa, Mohamed M. Abdel-Daim

**Affiliations:** ^1^ Department of Pharmacy, Southeast University, Dhaka, Bangladesh; ^2^ Pharmakon Neuroscience Research Network, Dhaka, Bangladesh; ^3^ Department of Pharmacy, Brac University, Dhaka, Bangladesh; ^4^ Teaching and Research Division, School of Chinese Medicine, Hong Kong Baptist University, Kowloon, Hong Kong; ^5^ Department of Pharmacy, Noakhali Science and Technology University, Noakhali, Bangladesh; ^6^ Institute of Health and Biomedical Innovation, Translational Research Institute, Brisbane, QLD, Australia; ^7^ School of Clinical Sciences, Queensland University of Technology, Brisbane, QLD, Australia; ^8^ Department of Pharmacy, BGC Trust University Bangladesh, Chittagong, Bangladesh; ^9^ Department of Biology, Faculty of Sciences, University of Hafr Al Batin, Hafr Al Batin, Saudi Arabia; ^10^ Department of Chemistry, University of Swabi, Khyber Pakhtunkhwa, Pakistan; ^11^ Department of Biology, College of Science, Princess Nourah bint Abdulrahman University, Riyadh, Saudi Arabia; ^12^ Zoology Department, Faculty of Science, Cairo University, Giza, Egypt; ^13^ Pharmaceutical Research Institute, Albany College of Pharmacy and Health Sciences, Rensselaer, NY, United States; ^14^ Department of Pharmaceutical Sciences, Pharmacy Program, Batterjee Medical College, Jeddah, Saudi Arabia; ^15^ Pharmacology Department, Faculty of Veterinary Medicine, Suez Canal University, Ismailia, Egypt

**Keywords:** NF-κB, glioblastoma, brain cancer, malignant, natural products, small molecules

## Abstract

Nuclear factor-κB (NF-κB) is a transcription factor that regulates various genes that mediate various cellular activities, including propagation, differentiation, motility, and survival. Abnormal activation of NF-κB is a common incidence in several cancers. Glioblastoma multiforme (GBM) is the most aggressive brain cancer described by high cellular heterogeneity and almost unavoidable relapse following surgery and resistance to traditional therapy. In GBM, NF-κB is abnormally activated by various stimuli. Its function has been associated with different processes, including regulation of cancer cells with stem-like phenotypes, invasion of cancer cells, and radiotherapy resistance identification of mesenchymal cells. Even though multimodal therapeutic approaches such as surgery, radiation therapy, and chemotherapeutic drugs are used for treating GBM, however; the estimated mortality rate for GBM patients is around 1 year. Therefore, it is necessary to find out new therapeutic approaches for treating GBM. Many studies are focusing on therapeutics having less adverse effects owing to the failure of conventional chemotherapy and targeted agents. Several studies of compounds suggested the involvement of NF-κB signaling pathways in the growth and development of a tumor and GBM cell apoptosis. In this review, we highlight the involvement of NF-κB signaling in the molecular understanding of GBM and natural compounds targeting NF-κB signaling.

## Introduction

In the United States, around 17,000 malignant glioma incidents are identified yearly, and more than 80% of these are glioblastoma multiforme (GBM) (Dolecek et al., 2012). GBM is characterized by a remarkably heterogeneous and invasive kind of malignant brain cancer (Uddin et al., 2020a). It is considered the deadliest cancer, with a projected survival of about 14 months after confirming the diagnosis (Furnari et al., 2007). Resistance to conventional therapies and tumor relapse after surgery are mainly responsible for this poor prognosis. GBM is highly heterogeneous and shows various distinctive properties with the unique origin of cell types and different genetic lesions lead to various clinical behaviors (Stiles and Rowitch, 2008; Huse and Holland, 2010). Current developments in molecular technology, primarily next-generation sequencing and high-density microarrays, have made it possible to classify GBM into subtypes according to the histological level and signatures of gene expression. Although many studies have tried to describe the various molecular subtypes of GBM (Phillips et al., 2006; Cooper et al., 2010; Verhaak et al., 2010; Huse et al., 2011), among them two subtypes, called proneural and mesenchymal (MES), tend to be stable and normally consistent amid the various proposed classifications. Furthermore, tumors identified as subtype MES demonstrate poor prognosis and have been associated with poor response to radiation, overexpression of CD44, and activation of nuclear factor-κB (NF-κB) (Bhat et al., 2013). There is still an inadequate comprehending of the fundamental processes of development and the reappearance of gliomagenesis, despite the advancement in genetic analysis and GBM classification.

NF-κB belongs to a family of transcription factors that leads to the formation of various heterodimers or homodimers and attach to consensus DNA sequences at the promoter regions of target gene (Friedmann-Morvinski et al., 2016). NF-κB plays a pivotal role in the control of immunity, inflammation, and cell survival, as well as involves in various functions associated with cellular activities (Tilstra et al., 2011; Uddin et al., 2021). Activation of NF-κB pathways could be triggered by different stimuli, such as DNA damage, cytokines, oncogenic stress, ultraviolet and ionizing radiation, pathogen-related molecular patterns, reactive oxygen species, and growth factors (Xia et al., 2014). Even though NF-κB has been revealed to develop cancer by triggering and maintaining a pro-inflammatory microenvironment, however; constitutive activation of NF-κB seems to induce tumor initiation and progression through several mechanisms, including apoptosis, proliferation of cells, metastasis of tumor, angiogenesis, and metabolism reprogramming (Xia et al., 2014). GBM has revealed that the constitutive NF-κB activation induces development and survival. Moreover, sulfasalazine (an anti-inflammatory drug) has shown inhibitory action of NF-κB that triggers the apoptosis in glioma cells (Robe et al., 2004), and a decoy oligonucleotide strategy to prevent NF-κB that played a crucial role in the decline in the number of cells (Gill et al., 2002). Recently, the activation of NF-κB has been found to connect with the transformation of MES differentiation from proneural glioma stem cells, with related radioresistant properties.

The natural compounds-based treatment has paid attention in scientists in the last 2 decades as an efficient and possibly less toxic therapy for cancer (Kumar et al., 2020; Lin et al., 2020; Aziz et al., 2021; Huang et al., 2021). Long years ago, American Indians had used *Podophyllum peltatum* L. roots for the treatment of various skin cancers (Mann, 2002). Furthermore, the key anticancer ingredient podophyllotoxin and its semi-synthetic derivatives, such as etoposide, etopophos, and teniposide are used to treat many cancers (Schacter, 1996). From 1960 to 1985, two mega-scale anti-cancer drug-screening projects were launched by the National Cancer Institute (NCI) (Cragg et al., 1996). After screening, they have found a significant compound paclitaxel (Taxol), separated from the *Taxus brevifolia* Nutt. bark, which has subsequently been applied in the treatment of several solid tumors (Cragg et al., 1996). Furthermore, almost one-third of the therapeutic agents accepted for cancer by the Food and Drug Administration (FDA) were from natural compounds or their derivatives (Mann, 2002; Altmann and Gertsch, 2007). Plant flavonol quercetin inhibits NF-κB transactivation in the U87 human glioma cell line at 20 µM and demonstrates antiproliferative effects, necrosis/apoptosis activation, and cell cycle arrest in the U138MG human glioma cell line with lower cytotoxicity to normal cells (Braganhol et al., 2006; Braganhol et al., 2007; Park and Min, 2011). Another study has suggested that by regulating NF-κB nuclear translocation and caspase-3 activation, a quercetin derivative causes cell death in glioma cells (Kiekow et al., 2016a). In this review, we focus on the role of NF-κB as a potential biomarker of GBM and highlight various natural compounds that affect the signaling pathway of NF-κB for treating GBM.

## Structural and Functional Properties of NF-κB

**FIGURE 1:**
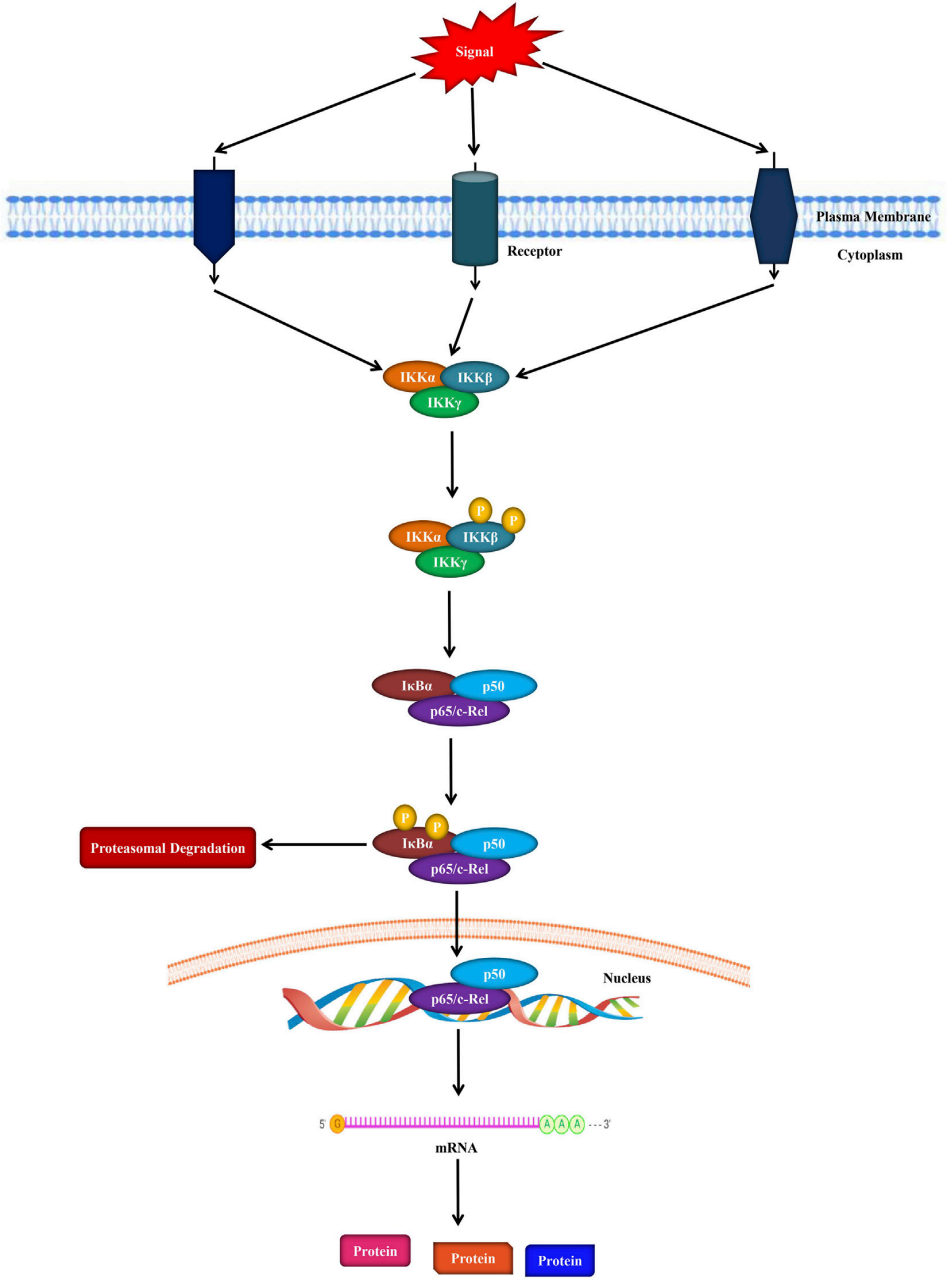
Schematic representation of the NF-κB signaling in cells.

NF-κB is a protein complex that regulates DNA synthesis, survival, and cytokine production (Taniguchi and Karin, 2018). Indeed, all the proteins that belong to the NF-κB family possess the Rel homology domain in their N-terminal part. In terms of its structure, NF-κB contains homodimers and heterodimers of the five members Rel family that are categorized into two groups. Furthermore, the first group of the protein complex is composed of c-Rel, RelA (p65), and RelB; on the other hand, the next group comprises NF-κB1 (p105/p50) and NFκB2 (p100/p52) (Sun, 2011; Puliyappadamba et al., 2014) as shown in Figure 1. It has been reported that the first group (c-Rel, RelA, and RelB) proteins contain a transactivation domain in their C-terminus region. Alternatively, second group specifically NF-κB1 and NF-κB2 proteins are generated as large precursors comprising p105 and p100 that play crucial roles in producing mature NF-κB subunits such as p50 and p52, successively. These NF-κB subunits have no inherent capacity to mediate transcription, and when they interact as homodimers κB components, they act as transcriptional repressors (Park and Hong, 2016).

It is known that kappa B (IκBs) inhibitors are a group of related enzymes with an N-terminal structural area, along with six or more ankyrin chains as well as a PEST domain adjacent to their C terminal domain. Even though the IκB family is composed of various proteins including Bcl-3, IκBα, IκBβ, and IκBε, among them IκBα has been extensively studied (Zhou et al., 2005). NF-κB is inactive in unstimulated cells due to its interaction with IκBα inhibitor, and the structure is usually found in the cytoplasm. IκB kinases (IKKβ or IKKα) are induced and phosphorylate IκBα in response to various inducers including DNA damage, vascular endothelial growth factor (VEGF), epidermal growth factor (EGF), and cytokines [i.e., tumor necrosis factor (TNF)-α and TRAIL] that can further result in its degradation via a K48 ubiquitin-induced proteasomal mechanism (Park and Hong, 2016).

Several stimuli may induce the mammalian NF-κB signaling pathways, such as growth factors, reactive oxygen species (ROS), oncogenic stress, DNA damage, pathogen-associated molecular patterns, ionizing and ultraviolet irradiation, various cytokines (TNF–α and IL-1β), and stress (Gilmore, 2006; Sun, 2011). It has been revealed that NF-κB is associated with many processes including inflammation, chemoresistance, cell survival, angiogenesis, metastasis, tumor progression, invasion, cell cycle progression, immunity, metabolic reprogramming, and apoptosis (Dolcet et al., 2005; Grivennikov and Karin, 2010). Increased levels of inflammatory cytokines (for example, IL-1, IL-15, IL-11, IL-6, and IL-8), C–C motif chemokine ligand (CCL)-2, and genes associated with pathological processes such as cyclooxygenase (COX)-2, cell adhesion (CD44), cell cycle modulators (Cyclin D1), and proteolysis (TFPI2, PLAU) are all known targets of the NF-κB signaling pathway (Richmond and Yang, 2016). Along with the nuclear translocation, several regulatory mechanisms, such as nuclear export processes, protein-protein interactions located in certain gene regulatory regions, and post-translatory changes of specific subunits of NF-κB, are controlled by the NF-κB signaling pathway. As a result, various cellular responses to NF-κB are frequently caused by specific and complex pathways that vary in their upstream stimuli and/or downstream targets (Gilmore, 2006).

## NF-κB in Glioblastoma

In case of GBM and other cancers, the NF-κB signaling pathway is found to be activated and is associated with a higher grade of astrocytic tumors. Characteristics of the mesenchymal subtype of GBM include increased concentrations of NF-κB signaling pathway constituents such as TNF receptor superfamily member 1A [(TNFRSF1A), v-rel reticuloendotheliosis viral oncogene homolog B (RELB), and TNFR1-associated death domain protein (TRADD)], elevated resistance towards chemotherapy and a lesser prognosis as compared to individuals with other GBM types (Verhaak et al., 2010). It has been established that an NF-κB/TNF- (tumor necrosis factor)-mediated mechanism can differentiate proneural individual-derived neurospheres to a mesenchymal phenotype, which has been linked to poor prognosis and radioresistance (Bhat et al., 2013). These findings collectively suggest the significance of the NF-κB signaling during the development and advancement of glioma. Applying gliomas as an experimental model for network analyses, it was observed that NF-κB signaling pathway dysregulation was 1 of 4 signaling mechanisms whose perturbation was slightly satisfactory to maintain the phenotype of malignant glioma (Karlebach and Shamir, 2010).

Nonetheless, the specific mechanism(s) of *in vivo* NF-κB activation in case of GBM is still unidentified. However, various proteins and signaling mechanisms are dysregulated in the case of GBM which might result in NF-κB activation. TNF-α is a very strong NF-κB activator (Grivennikov and Karin, 2011). TNF-α is a pro-inflammatory chemical that is released by astrocytes, microglia, and certain neurons in the CNS. Indeed, TNF-α exhibits its effect *via* two receptors including TNF receptor 1 (TNFR1) and TNF receptor 2 (TNFR2) (Grivennikov and Karin, 2011). Usually, TNFR1 is expressed in most type of the cells, whereas TNFR2 is expressed in oligodendrocytes and immune cells, including microglia. Furthermore, TNFR1 expression was found to be higher in GBM and GBM-linked endothelial cells than in normal brain tissues or low-grade gliomas (Hayashi et al., 2001; Kargiotis et al., 2006; Huang et al., 2012a).

Many signaling pathways or growth factors that are dysregulated in case of gliomas may result in the activation of NF-κB (Nogueira et al., 2011a). Particularly, NF-κB is activated *via* epidermal growth factor (EGF), and/or its receptor, (EGFR). It has been observed that EGFR is often mutated and constitutively activated (Bonavia et al., 2012; Yang et al., 2012; Puliyappadamba et al., 2013). Furthermore, oncogenic EGFR is associated with NF-κB activation by means of mechanistic target of rapamycin complex 2, and this signaling pathway mediates chemoresistance (Tanaka et al., 2011). Phosphatase and tensin homolog (PTEN) is a suppressor of tumor and a negative regulator of the Akt signaling pathway (Atkinson et al., 2010). It has been found that numerous gliomas exhibited loss of PTEN function (Atkinson et al., 2010). Akt is constitutively activated in case of PTEN absence, which can further result in NF-κB activation. Increased insulin-like growth factor binding protein-2 (IGFBP-2) concentrations and the signaling mechanism were found to be connected with an increased level of NF-κB signaling pathway (Holmes et al., 2012). In case of GBM, IGFBP2 binds specifically with β1 integrin in order to activate an integrin-linked kinase (ILK)/NF-κB cascade that further mediates the growth of glioma.

Various findings have suggested that abnormal expression of regulators can lead to increased activation of NF-κB. In GBM, the inhibitor of growth family member 4 (ING4, a negative NF- κB regulator) is expressed at very low concentrations or is mutated. In addition, deficiency of the ING4 effect activates NF-κB to remain active (Nozell et al., 2008). In contrast, peptidyl-prolyl isomerase (Pin1, a positive NF-κB regulator) was found to be overexpressed in case of GBM and plays role in constitutive activation of NF-κB (Atkinson et al., 2009). Plant homeodomain finger protein 20 (PHF20, an effector protein that binds with methylated p65) is a newly identified NF-κB regulator in GBM, which averts phosphatase PP2A recruitment, therefore it prolongs the presence of an active species of NF-κB in case of GBM (Zhang et al., 2013). It has been reported that the NFKBIA gene (that encodes IκBα) shows mono-allelic deletions in some GBM types, which is often lost in a subtype-specific manner in GBM, and this associates with increased levels of NF-κB as well as inferior prognosis of patients (Bredel et al., 2011). In a study, Pantane et al. (2013) confirmed that deletions of NFKBIA were elevated when tumors were spread as neurospheres in comparison with the deletions of parent tumor, which is further indicating that NFKBIA loss can mediate tumor-propagating activities and the formation of neurosphere. Collectively, these findings suggest the significance of the NF-κB signaling pathway in the stem cells of glioblastoma (Hjelmeland et al., 2010; Nogueira et al., 2011b).

Although many microRNAs (miRs) were found to be dysregulated and associated with the clinical-pathological characteristics of gliomas, but few of these were related to the NF-κB signaling pathway (Hummel et al., 2011). Oncogenic miRs (including miR-30e, miR-182, and miR-21) were linked with the NF-κB signaling pathway in GBM. Furthermore, miR-21 levels were much higher than in non-cancerous brain tissue in GBM, and were inversely related to patient prognosis. miR-21 levels were markedly increased as compared to non-cancerous brain tissue in GBM and inversely associated with the prognosis of patients (Chan et al., 2005; Ciafrè et al., 2005; Chen et al., 2008; Papagiannakopoulos et al., 2008; Lakomy et al., 2011). In GBM, overexpressed miR-21 induces NF-κB signaling pathway via targeting the leucine-rich repeat flightless-1-interacting protein 1 (LRRFIP1, a DNA-binding protein that inhibits the NF-κB signaling pathway) (Li et al., 2009). In terms of miR-182, around 98% of gliomas showed increased levels of miR-182. Moreover, the number of miR-182 copy was increased by 2–3 folds in approximately 35.6% glioma cases (Song et al., 2012). TGF-β increases miR-182 levels in GBM, which further mediates NF-κB signaling pathway *via* targeting various negative regulators of NF-κB, such as TNF-α-induced protein 3-interacting protein 1 (TNIP1), optineurin (OPTN), ubiquitin specific peptidase 15 (USP15), and cylindromatosis (CYLD). As compared to normal brain, the miR-30e level was increased in GBM and sustained NF-κB signaling pathway through the targeting of IκBα (Jiang et al., 2012).

Only a few tumor suppressive miRs have been related to the NF-κB signaling pathway in GBM until now. Some studies have been reported that miR-31 is mainly absent or downregulated in GBM (Hua et al., 2012; Wang et al., 2014) and its existence is found in both the classical and mesenchymal subtypes of GBM. Decreased levels of miR-31 are also a part of 10 miR expression feature that predicts patient survival independently (Srinivasan et al., 2011). It was revealed that miR-31 inhibits the NF-κB signaling pathway by targeting TRADD (a protein that plays a role as an upstream activator). In addition, both increased TRADD levels and miR-31 loss have been identified in case of mesenchymal subtype of GBM. Indeed, the aforesaid miRs offer an interesting miRs sampling which play role in NF-κB regulation, however the list of such miRs is quite lengthy and increasing (Song et al., 2013; Xia et al., 2013; Yang et al., 2014).

## Preclinical Studies of NF-κB Signaling in Glioblastoma

Still, it is challenging to target NF-κB for GBM. Furthermore, there is also a deficiency of specific and effective compounds. Nonetheless, bortezomib is a promising proteasome inhibitor that hinders the breakdown of IκBα as well as other proteins (Yin et al., 2005; Phuphanich et al., 2010). In addition, phase I clinical trials were also started for evaluating the maximum tolerated dose and side effects of bortezomib (Phuphanich et al., 2010). Even though that study did not provide extensive data regarding the efficacy of bortezomib in GBM, but there were some findings to suggest its clinical efficacy (Phuphanich et al., 2010). However, bortezomib is not yet considered a single-agent therapy for GBM treatment. In recent times, BAY-11 (an inhibitor of IKK) has been identified as the eminent NF-κB signaling pathway inhibitor. This inhibitor also mediated *in vivo* and *in vitro* senescence of GBM cells, ameliorated sensitivity towards photodynamic (5-ALA) therapy, and reversed chemoresistance (Coupienne et al., 2011; Nogueira et al., 2011b; Shukla et al., 2013). Interestingly, dehydroxymethylepoxyquinomicin (DHMEQ) has been identified as a unique small molecule NF-κB inhibitor (Fukushima et al., 2012). It has been confirmed by preclinical studies that DHMEQ inhibited activation of NF-κB and its nuclear translocation, resulting in decreased tumor growth *in vivo* as well as reduced proliferation in GBM cells *in vitro*. Furthermore, treatment with DHMEQ was found to be synergized with radiation and temozolomide, which is suggesting its significant therapeutic potential (Brassesco et al., 2013). It has also been confirmed that withaferin A (an inhibitor of IKKβ) significantly inhibits NF-κB signaling and GBM growth *in vivo* (McFarland et al., 2013). Even though withaferin A is presently being studied in a schizophrenia clinical trial (ClinicalTrials.gov Withan, 1793) and its extract for endurance exercise performance (ClinicalTrials.gov The Ef, 2021) but it is not under consideration for GBM treatment.

## Natural Products Targeting NF-κB Signaling in Glioblastoma

### Resveratrol

**FIGURE 2:**
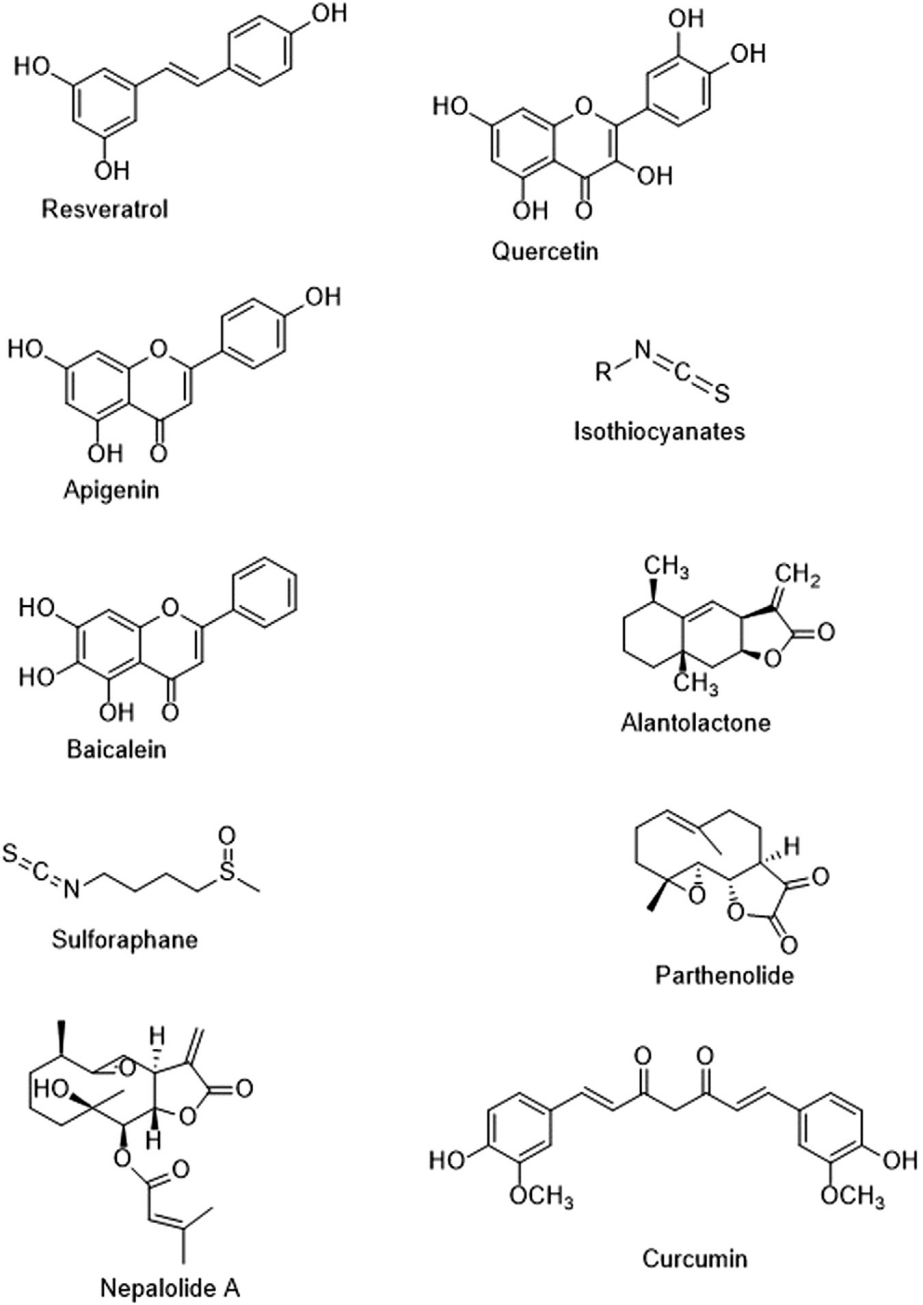
Chemical structures of the discussed molecules.

Resveratrol (a stilbenoid, naturally occurring polyphenolic compound, Figure 2) exhibited anti-invasion, immunomodulation, anti-inflammatory, and antitumor properties in several cancer cells (El-Readi et al., 2019; Jeandet et al., 2021). Numerous experiments have confirmed that resveratrol significantly supresses the NF-κB signaling *via* suppressing the activities of IκB kinase and NF-κB, which has provided a novel approach for cancer treatment (Ren et al., 2013; Vervandier-Fasseur and Latruffe, 2019). In a study, Jiao et al. (2015) confirmed that resveratrol suppressed PI3K/Akt/NF-κB signaling cascade that led to the inhibition of matrix metalloproteinase (MMP)-2 expression, which further resulted in suppression of attack in GBM-initiating cells. Furthermore, it was confirmed that there is a connection between the NF-κB effect and invasiveness of GBM (which takes place because of the processing of fibronectin *via* MMPs), which allows the direct integration of MMPs into the surrounding tumor cells (Westhoff et al., 2013). In addition, in GBM cells (T98G), resveratrol reversed the temozolomide resistance *via* O-6-methylguanine-DNA methyltransferase (MGMT) downregulation *via* the NF-κB-dependent pathway (Huang et al., 2012b). Many studies suggested that the NF-κB activation in multiple tumor cells (primarily associated with drug resistance), which was found to be facilitated *via* numerous chemotherapy medicines and radiation because of its action in MGMT transcription (Li and Sethi, 2010). Interestingly, NF-κB-p65 (an NF-κB subunit) resulted in increased MGMT expression, whereas an inhibitor of NF-κB abolished the increased expression of MGMT. (Hegi et al., 2005; Arepalli et al., 2015). Therefore, targeting MGMT, IκB kinase, and NF-κB may be beneficial in the resveratrol’s anti-GBM potential.

### Quercetin

Quercetin (a plant flavonoid, Figure 2) can induce cell death of brain, liver, and breast cancer cells (Anand David et al., 2016). Furthermore, this phenolic compound shows antioxidant, neuroprotective, anti-inflammatory, anticarcinogenic, and antihypertensive properties (Reyes-Farias and Carrasco-Pozo, 2019; Zaplatic et al., 2019). It has been revealed that quercetin has the capacity to regulate several protein kinases, particularly the PI3K pathway (Liu et al., 2017a). In a study, Liu et al. (2017b) studied the activity of quercetin (10 μg/ml) on cell invasion and migration. These researchers observed that a low dose of quercetin suppressed *in vitro* angiogenesis and glioblastoma cell invasion. In addition to this, they reported that quercetin (10 μg/ml) suppressed cell migration and human umbilical vein endothelial cell tube formation mediated via the U251 cell culture-derived conditioned medium. This suppressive action of quercetin on angiogenesis and migration might be induced by the downregulation of protein concentrations of MMP-2, MMP-9, and vascular endothelial growth factor (Liu et al., 2017b). In a study, Kiekow et al. (2016b) revealed that quercetin stimulates apoptosis in GBM cells *via* regulating activation of caspase-3 and nuclear translocation of NF-κB, which is further indicating the potential of quercetin as a novel anti-GBM therapy. Phospholipase D (PLD) overexpression induced MMP-2 expression. Therefore, GBM cell invasion by protein kinase A (PKA)/NF-κB and protein kinase C-induced signaling mechanisms (Park et al., 2009; Tang et al., 2017). Furthermore, it was shown that quercetin suppressed the NF-κB-mediated expression of PLD-1 by the mitigation of NF-κB transactivation (Park and Min, 2011).

### Apigenin

Apigenin is a natural flavone, (Figure 2) which is mostly found in tea leaves and fruits exhibits several biological properties including anticancer (prostate, liver, breast, and lung cancers), anti-inflammatory, antiviral, cytotoxic, antioxidant, and immunoregulatory properties (Erdogan et al., 2016; Yan et al., 2017). Apigenin’s immunoregulatory property is induced *via* the suppression of PI3K/Akt/NF-κB signaling cascade (control of IKK and IκBα) signaling pathways in various cancers (Chang et al., 2015; Erdogan et al., 2016; Qin et al., 2016), which can further result in a decreased level of metastasis and invasion. Chen et al. (2016) confirmed that apigenin in a dose-dependent manner markedly reduced cell viability and triggered apoptotic cell death of U87 cells. In U87 cells, it was confirmed that apigenin markedly elevated the levels of microRNAs (miR)-16, inhibited the NF-κB/MMP-9 pathway, and also inhibited the expression of BCL2 protein. Interestingly, anti-miR-16 plasmid-mediated miR-16 downregulation was found to reverse the activity of apigenin on NF-κB/MMP-9 signaling pathway, expression of BCL2 protein, and cell viability. Collectively, findings of the study indicated that apigenin suppresses glioma cell growth *via* inducing miR-16 and inhibiting NF-κB/MMP-9 and BCL2 (Chen et al., 2016).

### Isothiocyanates

Isothiocyanates (Figure 2) are natural compounds derived from the glucosinolate precursors of cruciferous vegetables, which exert anticancer, anti-invasion, and anti-inflammatory actions *via* inhibiting breast cancer, pancreatic cancer, and myeloma (Prawan et al., 2009; Brunelli et al., 2010). Many studies have reported that the anti-inflammatory effects of isothiocyanates in multiple cancer cells (Subedi et al., 2017; Mitsiogianni et al., 2019). The consequences have indicated that the anti-inflammatory properties of isothiocyanates may be mediated by the inhibition of c-Jun N-terminal kinase (JNK)/NF-κB/TNF-α signaling pathways (Subedi et al., 2017). In a study, Guo et al. (2019) assessed the possible synergistic action of phenethyl isothiocyanate in combination with temozolomide and theorized that phenethyl isothiocyanate might facilitate the anticancer role of temozolomide partly *via* NF-κB-dependent signaling pathway reducing MGMT expression. Furthermore, treatment with phenethyl isothiocyanate elevated the sensitivity of temozolomide resistant U373, U87, and T98 cells *via* suppressing the MGMT expression through the NF-κB signaling cascade (Guo et al., 2019). In order to examine the inhibitory effects of isothiocyanates on cancer invasion and migration, Lee et al. (2015) looked at isothiocyanate-regulated MMP-9 activation in C6 glioma cells. MMP-9 is a crucial enzyme in cancer metastasis that destroys the extracellular matrix. They revealed that isothiocyanates suppressed MMP-9 transcription levels *via* inhibiting NF-κB and activator protein-1 (AP-1), which further preventing C6 GBM cell motility and invasion (Lee et al., 2015).

### Sulforaphane

Sulforaphane (a natural isothiocyanate group of organosulfur compounds, Figure 2) is derived from broccoli sprouts (Uddin et al., 2020b). Sulforaphane shows cancer-protective effects *via* detoxifying and ameliorating antioxidant capacity (Clarke et al., 2008; Cheung and Kong, 2010). In human cancer cells, sulforaphane-mediated inhibition of NF-κB and NF-κB-regulated gene expression takes place *via* IκBα and IKK signaling pathways (Xu et al., 2005). Sulforaphane altered caspase-9/-12 cleavage, cytochrome C release, calpain effect, DNA fragmentation, intracellular Ca^2+^ level, morphological characteristics, Bcl-2-associated X-protein (Bax)/B-cell lymphoma 2 (Bcl-2) ratio, and levels of IκBa protein in U-87 MG and T98G GBM cells (Lenzi et al., 2014). In GBM cells, sulforaphane suppressed the inhibitor of apoptosis proteins and IκBα up-regulation, resulting in downregulation of NF-κB expression (Huang et al., 2012c). Lan et al. (2016) showed that sulforaphane might markedly inhibit proliferation of temozolomide-resistant GBM cells. Sulforaphane also suppressed the function of NF-κB pathway and then decreased the expression of MGMT in order to reverse the chemo-resistance to temozolomide in U373-R, U87-R, and T98G cell lines. Moreover, sequential combination with temozolomide synergistically elevated apoptosis induction and suppressed survival capability in temozolomide-resistant GBM cells. It was also suggested that sulforaphane might significantly increase cell death and inhibit cell growth in a chemo-resistant xenograft nude-mouse model (Lan et al., 2016). In a different study, Li et al. (2014) observed that sulforaphane suppresses invasion *via* inducing activation of ERK1/2 signaling pathway in human glioblastoma U373MG and U87MG cells. Bijangi-Vishehsaraeiv et al. (2017) confirmed that sulforaphane markedly suppresses GBM cell survivals and triggers apoptosis in GBM cells linked with the elevated activity of caspase-3 and -7. In addition, sulforaphane did not influence normal human mesenchymal stromal cells and exerted modest action on the nontumor brain cells.

### Alantolactone

Alantolactone, a sesquiterpene lactone extracted from *Inula helenium* L. (Figure 2), has many pharmacological properties, including anticancer, antibacterial, antifungal, and anti-inflammatory properties (Chun et al., 2012). Alantolactone’s antitumor activities were observed in chronic myelogenous leukemia, brain tumors, colorectal cancer, lung cancer, and liver cancer (Khan et al., 2012; Rasul et al., 2013; Wei et al., 2013). In GBM cells, alantolactone caused cell death through glutathione depletion, mitochondrial dysfunction, and ROS production (Khan et al., 2012). Furthermore, alantolactone-induced other mechanisms triggered involve suppression of inducible nitric oxide synthase (iNOS) and COX-2 expression and downregulation of NF-κB and AP-1 through myeloid differentiation primary response 88 (MyD88) signaling (Chun et al., 2012). In a study, Wang et al. (2017) revealed that alantolactone’s antitumor activity against GBM. This study found that the alantolactone dramatically inhibited the growth of GBM. Alantolactone inhibited the kinase activity of IKKβ by targeting the ATP-binding site and then attenuated the binding of NF-κB to the COX-2 promoter area, resulting in a considerable reduction in COX-2 expression. Alantolactone also triggered apoptosis by activating the caspase/cytochrome c signaling pathway. Furthermore, alantolactone can penetrate the blood-brain barrier (BBB). Thus anti-tumor effects of alantolactone is facilitated by blocking the activity of IKKβ kinase as well as affecting NF-κB/COX-2-induced signaling mechanisms in GBM (Wang et al., 2017). Therefore, due to its dual inhibitory effects on NF-κB and IKKβ expression, alantolactone may be considered a promising natural compound against GBM.

### Baicalein

Baicalein (a bioactive flavone, Figure 2) is derived initially from *Scutellaria baicalensis* Georgi and *Scutellaria lateriflora* L. (Varsha et al., 2017). This flavone has traditionally been utilized because of its anticancer activities (Liu et al., 2016; Zhao et al., 2016). In multiple cancer cell lines, aicalein inhibited the nuclear translocation of NF-κB and exerted anti-inflammatory activities (Seo et al., 2011; Yu et al., 2018a). Moreover, baicalein inhibited the C33A growth and accelerated cellular apoptosis by blocking NF-κB spathway (Yu et al., 2018a). After treatment with baicalein, NF-κB-p65 activity and expression were markedly suppressed in U251 GBM cells (Jiang et al., 2016). The use of an NF-kB-p65 inhibitor (EVP4593) combined with baicalein resulted in a synergistic reduction of Bcl-2 expression, followed by an increase in Bax and cleaved-caspase-3 expression, as well as the inhibition of U251 cell survival (Jiang et al., 2016). All of these finding indicating that baicalein might be therapeutically used as a natural compound against GBM.

### Parthenolide

Parthenolide (a naturally occurring sesquiterpene lactone, Figure 2) is extracted from Tanac*etum parthenium* L. (Chaturvedi, 2019). This naturally occurring compound inhibits NF-κB *via* suppressing the activity of IκB kinase as well as altering the p65 subunit (Kwok et al., 2001). Furthermore, parthenolide has traditionally been used to treat rheumatoid arthritis and migraine because of its anti-inflammatory properties and low toxicity (Heptinstall, 1988). In numerous studies, the effects of parthenolide on animal malignancies have been widely explored (Gilmore, 2006; Haffner et al., 2006). It was revealed that parthenolide inhibited proliferation, angiogenesis, and invasion of GBM cells (U87MG and U373). In a study, Yu et al. (2018b) revealed that the suppression of NF-κB resulted in anti-GBM effect and suppressed temozolomide-mediated chemoresistance *via* downregulation of *MGMT* gene expression. Furthermore, it has been confirmed by Nakabayashi and Shimizu (2012) that parthenolide inhibits angiogenesis and decreases phosphorylation of Akt and activated mitochondrial signaling pathway, which is suggesting that the antitumor effect of parthenolide might be induced *via* stimulation of apoptosis, suppression of Akt signaling pathway, and NF-κB suppression. In glioblastoma xenografts, parthenolide reduced neovascularity and tumor development (Nakabayashi and Shimizu, 2012). However, it has been observed that treatment with parthenolide causes rapid cell death *via* caspase-3/-7 without influencing the regulation of NF-κB in GBM cells (Anderson and Bejcek, 2008).

### Nepalolide A

Nepalolide A (a naturally occurring germacranolide sesquiterpene lactone, Figure 2) is derived from *Carpesium nepalense* Less. (Wang et al., 1999). In C6 glioma cells and primary astrocytes, the effects of nepalolide A on the production of iNOS generated by LPS/IFN-γ or TNF-α/IL-1β/IFN-γ were studied by Wang et al. (1999). This study reported that nepalolide A inhibited signaling pathways induced *via* cytokine and lipopolysaccharide and also suppressed the phosphorylation of IκB protein (Wang et al., 1999). Thus, suppression of NF-κB activation *via* nepalolide A was facilitated *via* blocking IκB degradation, which can further result in suppression of iNOS expression (Wang et al., 1999).

### Curcumin

Curcumin (a polyphenol, Figure 2) is the major bioactive compound of *Curcuma longa* L. that exerts anti-inflammatory and antioxidant properties by decreasing the activity of AP-1 and NF-κB (Saberi-Karimian et al., 2019). Its potential to treat various high-risk cancers has also been assessed in phase I clinical trials (Cheng et al., 2001). Furthermore, curcumin might exert numerous beneficial effects in GBM cells, such as suppression of angiogenesis, cell development, and invasion (Ambegaokar et al., 2003; Gao et al., 2005; Kim et al., 2005). Its activities on GBM development were also studied in the rat (C6) and human (T98G, T67, and U87MG) GBM cell lines. Furthermore, curcumin decreased the survival of cells in a p53-and caspase-independent manner, an activity which is linked with the suppression of NF-κB and AP-1 signaling pathways by the deterrence of constitutive Akt and JNK activation (Dhandapani et al., 2007). Curcumin also induced the antitumor effect of nimustine (a nitrosourea with antineoplastic activity) against GBM *via* inhibiting the NF-κB/COX-2 and PI3K/Akt signaling cascades (Zhao et al., 2017a). In a study, Fratantonio et al. (2019) reported that curcumin triggered the anti-GBM effect of paclitaxel by suppressing NF-κB activation in rat C6 cells. In U87 cells, demethoxycurcumin (a curcuminoid) exerted anti-proliferative effects *via* inhibition of the Akt/NF-κB signaling cascade (Kumar et al., 2018).

In a study, Wu et al. (2015) determined whether the cytotoxic effects of curcumin in glioblastoma cells is facilitated *via* miR-146a upregulation. Roles of curcumin and temozolomide (alone or in combination) on apoptosis and cell proliferation were assessed in human U-87 MG glioblastoma cells. Curcumin treatment resulted in miR-146a upregulation in U-87 MG cells. As compared to single treatment, combined treatment curcumin and temozolomide markedly (*p* < 0.05) stimulated apoptotic death and suppressed proliferation of U-87 MG cells. Depletion of miR-146a blocked the curcumin-induced enhancement of temozolomide-mediated apoptosis (Wu et al., 2015). Overexpression of miR-146a inhibited activation of NF-κB and increased apoptosis in temozolomide-treated cells. Furthermore, pharmacological suppression of NF-κB signaling pathway markedly elevated temozolomide-mediated apoptosis (Wu et al., 2015).

## Natural Molecules and Blood-Brain Barrier Permeability

The brain is incredibly equipped with a significant protective element known as the BBB. BBB plays a role in preventing harmful substances from entering the brain. Because of this property of BBB, most of the new drugs cannot enter into and treat the brain tumor. Furthermore, BBB is one of the major challenges that researchers need to overcome to develop novel and effective treatments for individuals with malignant brain tumors (Noch et al., 2018). Indeed, BBB regulates the entry of various molecules into the brain *via* transcellular or paracellular signaling pathways. It has been reported that BBB also contains many tight junctions between endothelial cells, ATP-dependent multidrug resistance pathway proteins called P-glycoprotein (P-gp), enzymes, and receptors (Chow and Gu, 2015). Components of the BBB microenvironment include extracellular matrix, basement membrane, fibroblasts, neurons, pericytes, microglia, and astrocytes (Obermeier et al., 2013), which also affect BBB activities (Lécuyer et al., 2016; Zhao et al., 2017b). It has been identified that blood-brain tumor-barrier (BBTB) arises from tumor capillaries providing oxygen and nutrients to the tumor (Tellingen et al., 2015). On the other hand, the glioma BBTB microenvironment contains infiltrating macrophages, tumor-linked microglia, extracellular matrix, tumor cells, and various other types of cells. In rat GBM models, tumor growth was suppressed by targeting the glioma microenvironment (Jacobs et al., 2012). Targeted therapy has gained a lot of attention over time, but this therapy did not raise the overall survival of individuals with GBM, partly owing to the poor drug penetration across the BBB. Thus, there is a growing interest regarding the molecules that can modulate BBB permeability to ameliorate the bioavailability of therapeutic agents into tumors. Furthermore, nanotechnology is essential to mask the physicochemical properties of therapeutic agents to prolong half-life across the BBB (Gerstner and Fine, 2007).

In the case of brain cancer treatment, targeted delivery of resveratrol into the brain tumor tissue might overcome various issues, including chemical instability, poor water solubility, and low bioavailability of resveratrol. Multiple types of polymeric nanoparticles and liposomes have already been developed to enhance GBM treatment. In a study, Vijayakumar et al. (2016) showed that the antiglioma cytotoxicity, passive brain targeting, and biological half-life of resveratrol were markedly improved *via* utilizing d-α-tocopheryl polyethylene glycol-1000 succinate-coated liposomes. In another study, Guo et al. (2013) altered the surface of resveratrol-loaded polyethylene glycol-polylactic acid nanoparticles with transferrin moieties (Tf-NP), which further resulted in increased cytotoxicity, elevated level of intracellular uptake, and apoptosis of human U-87 MG GBM cell lines and C6 rat glioma cells *in vitro*, as compared to nanoparticles without transferrin and free resveratrol. As transferrin receptors are only expressed in brain capillaries (Johnsen et al., 2017), the buildup of Tf-NP-resveratrol in tumor tissues prolonged survival and reduced tumor volume in rat models containing C6 orthotopic glioma. In a study, Wei et al. (2015) observed that treatment with resveratrol protected BBB integrity *via* regulating activities and expressions of TIMP-1 and MMP-9 in brains of rat models that were reperfused after ischemic injury.

Quercetin was found to cross the BBB and decrease the advancement of degenerative diseases (Li et al., 2015). It has been suggested that *α*-tocopherol can mediate the transport of quercetin through the BBB. In the brain, the combination of quercetin and rutin mediated the buildup of quercetin and/or its conjugated derivatives (Ferri et al., 2015). Quercetin was found to improve BBB dysfunction, reduce BBB leakage, and decrease brain edema (Aziz et al., 2021). Quercetin may decrease the expressions of axin, GSK-3β, and MMP-9, and elevate the expression of β-catenin, claudin-5, and ZO-1. Interestingly, all these protective activities of quercetin might be reversed *via* DKK-1 (Aziz et al., 2021). Adhesion molecules, membrane proteins, or invasion-linked proteins may play roles in tumor invasion and migration. In human glioblastoma cells, sulforaphane-cysteine suppressed the invasion and migration by increasing the fusion of mitophagosome to the lysosome (Zhou et al., 2020). Collectively, these findings might help in developing low-toxicity and high-efficiency anticancer drugs to suppress invasion and migration in GBM. Wang et al. (2013) confirmed that curcumin has the ability to maintain the integrity of BBB *via* regulating the expressions of occludin and ZO-1 during glucose deprivation and hypoxia. Curcumin and curcumin-loaded PLGA nanoparticles penetrated the BBB to enter brain tissues, where it was accumulated primarily in the hippocampus. Nanotechnology markedly increased the retention time of curcumin in the hippocampus (elevated by 83%) and cerebral cortex (elevated by 96%) (Tsai et al., 2011). In brain microvascular endothelial cells, curcumin improved the permeability of BBB during hypoxia *via* upregulating the expression of heme oxygenase-1 (Phillips et al., 2006). All of the evidence warrant more preclinical and clinical studies for better understand the potential therapeutic benefits.

## Conclusion

Considerable efforts are being invested to develop antineoplastic agents, but these agents have various limitations, including severe side effects and high cost. Since there are several adverse effects of currently available inhibitors of NF-κB, thus investigating the currently available naturopathic formulations can provide a perfect platform for identifying novel inhibitors of NF-κB considering the specific and non-specific multi-target activities of phytochemicals on several constituents of the NF-κB signaling pathway. The activation of NF-κB ensures a pro-proliferative activity in GBM. Inhibiting this pathway *via* suppressing the NF-κB effect or specially NF-κB–inducible genes might be a promising therapeutic method to treat GBM. Numerous attempts have been made to include inhibition of NF-κB into GBM treatment, but no precise outcome has been attained so far. Even though some naturally occurring compounds that inhibit NF-κB signaling pathways (particularly IKK inhibitors) are available, but more specific inhibitors of IKK and other upstream kinases need to be further studied clinically to confirm their potential in patients with GBM. Various natural compounds contain auspicious NF-κB modulatory activities in the treatment of GBM. As NF-κB modulators exhibited low toxic effects against normal astrocytes (which indicate their selectivity towards cancer cells), hence phytochemicals might play a role as potential agents in clinical trials, facilitating the finding of better therapy to treat GBM. In GBM patients, most of the chemotherapy trials failed partially because of the poor drug penetration across the BBB. It has been observed that natural products might alter the permeability of BBB *via* modifying the action of its constituents. Most of these mentioned phytochemicals are yet to be studied in human clinical trials and/or cancer models for their anticancer properties, bioavailability, and biocompatibility. As compared to available synthetic inhibitors of NF-κB, these natural products possess several advantages, including efficacy and safety. Along with conventional cancer therapy, the use of these natural products as adjunctive chemotherapy might establish a better therapeutic approach. However, structural alterations of these phytochemicals ought to be evaluated to conceivably improve the antineoplastic property of these natural compounds. Enormous data are available to support more studies in utilizing natural compounds in GBM. Indeed, this poor prognosis requires the exploration of alternative therapeutic agents to ameliorate outcomes for the affected individuals. Prospective randomized trials are required to find the use of adjunctive natural therapies for the better targeting of resistance, to study safety profiles, and synergistically ameliorating existing therapies.
